# Differential Roles of Cysteinyl Cathepsins in TGF-β Signaling and Tissue Fibrosis

**DOI:** 10.1016/j.isci.2019.08.014

**Published:** 2019-08-09

**Authors:** Xian Zhang, Yi Zhou, Xueqing Yu, Qin Huang, Wenqian Fang, Jie Li, Joseph V. Bonventre, Galina K. Sukhova, Peter Libby, Guo-Ping Shi

**Affiliations:** 1 Department of Medicine, Cardiovascular Medicine, Brigham and Women's Hospital, Harvard Medical School, 77 Avenue Louis Pasteur, NRB-7, Boston, MA 02115, USA; 2 School of Food & Biological Engineering, Hefei University of Technology, Hefei 230009, China; 3 Department of Nephrology, First Affiliated Hospital, Sun Yat-Sen University, Guangzhou 510080, China; 4 Department of Rheumatology, Nanfang Hospital, Southern Medical University, Guangzhou 510515, China

**Keywords:** Fibrosis, Molecular Mechanism of Behavior, Functional Aspects of Cell Biology

## Abstract

Transforming growth factor beta (TGF-β) signaling contributes to tissue fibrosis. Here we demonstrate that TGF-β enhances CatS and CatK expression but reduces CatB and CatL expression in mouse kidney tubular epithelial cells (TECs). CatS- and CatK deficiency reduces TEC nuclear membrane importer importin-β expression, Smad-2/3 activation, and extracellular matrix (ECM) production. Yet CatB- and CatL-deficiency displays the opposite observations with reduced nuclear membrane exporter RanBP3 expression. CatS and CatK form immunocomplexes with the importin-β and RanBP3 more effectively than do CatB and CatL. On the plasma membrane, CatS and CatK preferentially form immunocomplexes with and activate TGF-β receptor-2, whereas CatB and CatL form immunocomplexes with and inactivate TGF-β receptor-1. Unilateral ureteral obstruction-induced renal injury tests differential cathepsin activities in TGF-β signaling and tissue fibrosis. CatB- or CatL-deficiency exacerbates fibrosis, whereas CatS- or CatK-deficiency protects kidneys from fibrosis. These cathepsins exert different effects in the TGF-β signaling cascade independent of their proteolytic properties.

## Highlights

• Cathepsins exert different activities in kidney TEC TGF-β signaling and fibrosis
• Cathepsins regulate nuclear membrane transporter expression and form immunocomplexes
• Cathepsins regulate plasma membrane TGF-β receptor expression and form immunocomplexes
• Cathepsins play different roles in acute injury-induced kidney fibrosis

## Graphical Abstract

**Figure:**
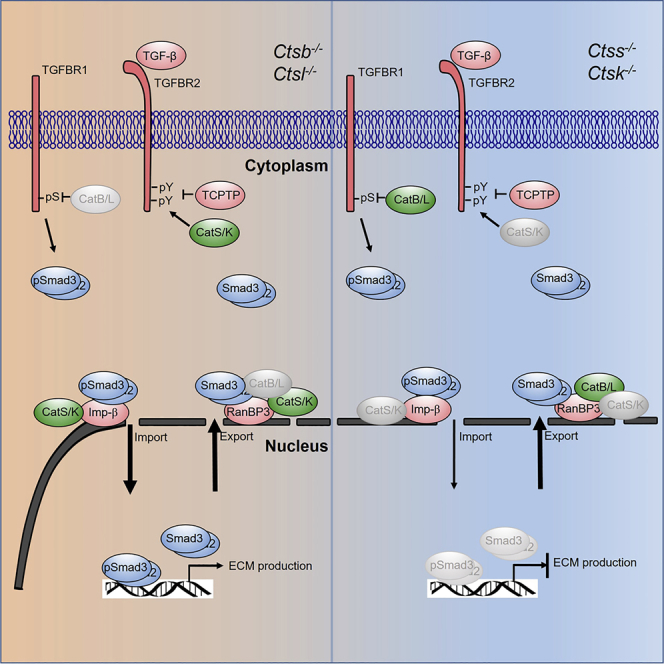

## Introduction

Transforming growth factor beta (TGF-β), a pleiotropic mediator of fibrotic remodeling, induces myofibroblast transition, Smad activation, and extracellular matrix (ECM) production; inhibits excessive ECM protein degradation by regulation of proteases and their endogenous inhibitors; and regulates integrin expression and cell adhesion to the matrix (Border and Noble, 1994, Lan, 2003). Active TGF-β binds to TGF-β receptor-2 (TGFBR2). This binding recruits and activates TGF-β receptor-1 (TGFBR1) (Massague, 1998), followed by phosphorylation of Smad-2 and Smad-3 (Roberts, 1998, Schnaper et al., 2002). Phosphorylated Smad-2 and Smad-3 (pSmad-2/3) and Smad-4 form a Smad complex (Feng and Derynck, 2005) that translocates to the nucleus to regulate the expression of profibrotic genes such as collagen, fibronectin, and α-smooth muscle actin (α-SMA) (Massague, 1998), leading to tissue fibrosis that characterizes diseases of multiple tissues and organs. Cells denoted myofibroblasts elaborate ECM in response to TGF-β and can arise from fibroblasts, endothelial cells, epithelial cells, or even macrophages (Allison, 2013, LeBleu et al., 2013, Meng et al., 2014, Nikolic-Paterson et al., 2014).

TGF-β signaling and Smad activation determine ECM production and tissue fibrosis. Lungs from patients with cystic fibrosis and idiopathic pulmonary fibrosis (IPF) show increased pSmad-2, the myofibroblast marker α-SMA, and collagen deposition (Harris et al., 2013). Transgenic expression of TGF-β augments macrophage-dependent lung fibrosis (Murray et al., 2011). Lung epithelial cell-specific depletion of TGFBR2 protects mice from bleomycin-induced fibrosis (Li et al., 2011). Elevated TGF-β in infarcted hearts correlates with impaired left ventricular function (Talasaz et al., 2013). Continued TGF-β activation promotes chronic hypertension, progressive myocardial fibrosis, and heart failure. Infarcted hearts from Smad-3-deficient mice show reduced fibrosis (Bujak et al., 2007), and fibroblasts from these mice do not respond to TGF-β (Verrecchia et al., 2001). Chronic liver damage-induced TGF-β activates hepatic stellate cell (HSC)-to-myofibroblast transition and hepatocyte death and promotes liver fibrosis, cirrhosis, and ultimately hepatocellular carcinoma (Dongiovanni et al., 2014). Chronic kidney diseases involve renal fibrosis that contributes to organ failure (Eddy and Neilson, 2006). TGF-β1 induces tubular epithelial cell (TEC)-to-myofibroblast transition and promotes renal fibrosis (Lan, 2003).

Cysteinyl cathepsins mediate ECM protein degradation and mitigate tissue fibrosis. TGF-β lowers cathepsin L (CatL) expression in lung epithelial cells (Gerber et al., 2001) and cathepsin K (CatK) expression in fibroblasts, favoring lung fibrosis (van den Brule et al., 2005). Deficiency of CatK or CatL exacerbates lung and myocardial fibrosis and cardiomyopathy (Buhling et al., 2004, Petermann et al., 2006), whereas their overexpression reduces ECM deposition, cardiac hypertrophy, and cardiac and lung fibrosis (Tang et al., 2009, Zhang et al., 2011a, Zhang et al., 2011b). These studies suggest a protective role of cathepsins in tissue fibrosis via their ECM degradation. Yet, fibroblasts in fibrotic lungs from patients with IPF express high levels of CatK. CatK inhibition decreases pSmad-2/3, diminishes α-SMA expression, and delays fibroblast differentiation (Buhling et al., 2004). In bleomycin-induced lung fibrosis, cathepsin activities increase in lung homogenates and in lavage fluid (Kasabova et al., 2016). Liver CatL and cathepsin B (CatB) levels also increase in patients with hepatic cirrhosis and in mice with CCl_4_-induced liver fibrosis (Manchanda et al., 2017). CatB inhibition or depletion reduces mouse liver inflammation and fibrogenesis (Moles et al., 2009, Moles et al., 2012). Deficiency of cathepsin S (CatS) reduces mouse myofibroblast differentiation and Smad activation and impairs post-infarct cardiac functions (Chen et al., 2013). Therefore, cathepsin activities beyond ECM degradation may contribute to tissue fibrosis.

This study used kidney TECs from mice deficient in CatB, CatL, CatS, and CatK and demonstrated that individual members of this highly related class of cathepsins contributed in opposing manners to TGF-β signaling, Smad activation, and profibrotic protein production. These distinct activities arose from differential regulation of cathepsin expression and interaction with and activation of nuclear membrane Smad complex transporter proteins (importin-β and RanBP3) and plasma membrane TGF-β receptors (TGFBR2 and TGFBR1). We validated these observations in fibrosis-dependent unilateral ureteral obstruction (UUO)-induced mouse renal injury, in which individual cathepsins showed opposite activities in renal Smad activation, epithelial cell differentiation, and kidney fibrosis.

## Results

### Differential Role of Cathepsins in TGF-β-Induced ECM Production in Mouse Kidney TECs

**Figure 1:**
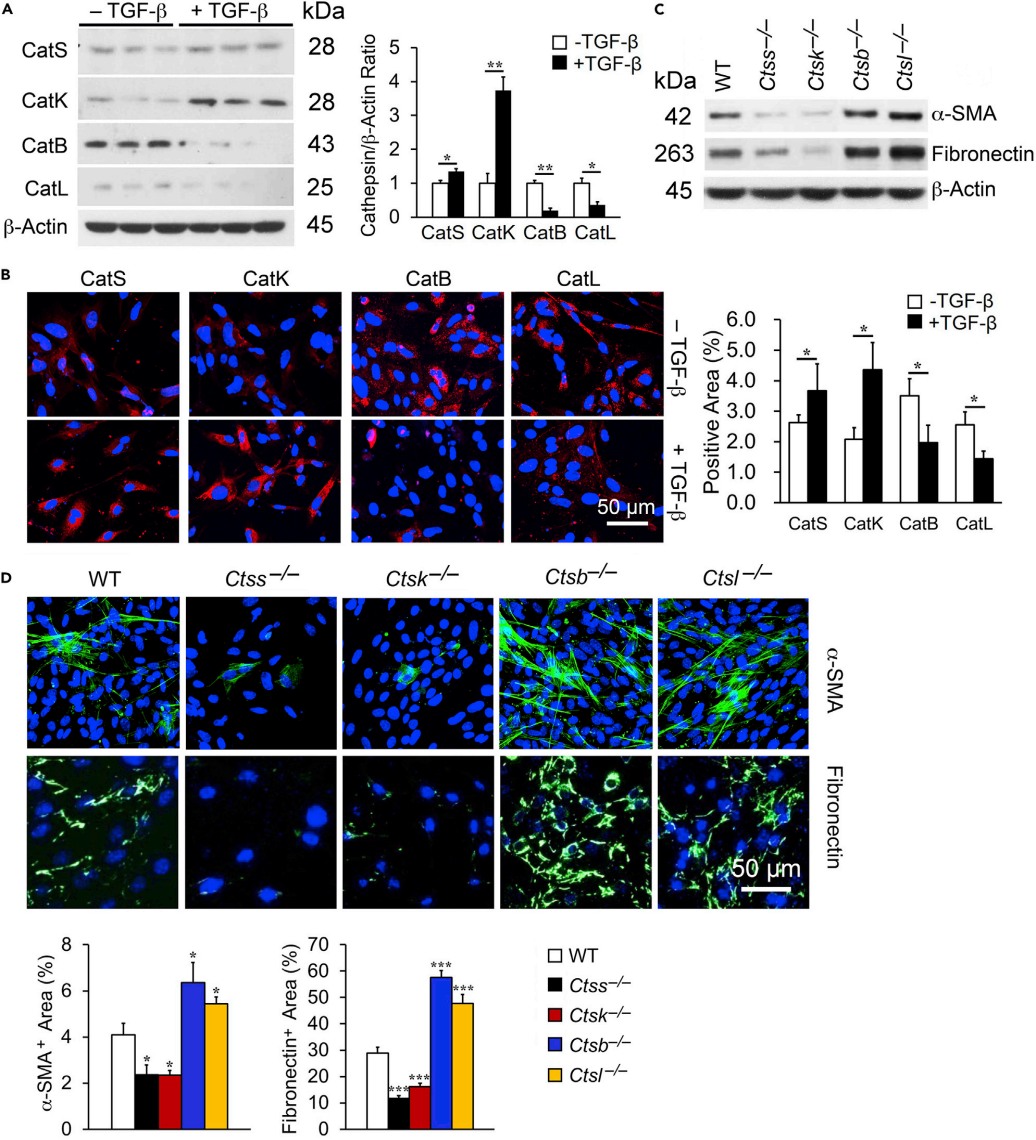
TGF-β-Regulated Expression of Cathepsins and Fibrotic Proteins in Mouse Kidney TECs (A–D) Immunoblot (A) and immunofluorescent staining detected the expression of CatB, CatL, CatS, and CatK (red) with nuclei counterstained with DAPI (blue) and quantification (B) in WT kidney TECs with or without 2 ng/mL TGF-β stimulation. Immunoblot (C) and immunofluorescent staining detected the expression of α-SMA (green) and fibronectin (green) with nuclei counterstained with DAPI (blue) and quantification (D) in TGF-β-treated (2 ng/mL, 24h) TECs from WT, *Ctsb*^*−/−*^, *Ctsl*^*−/−*^, *Ctss*^*−/−*^, and *Ctsk*^*−/−*^ mice. Scale bar: 50 μm. Data are representative of four independent experiments. ∗p < 0.05, ∗∗p < 0.01, ∗∗∗p < 0.001.

TGF-β promotes kidney TECs epithelial-mesenchymal-transition into myofibroblasts that release pro-inflammatory and profibrotic proteins during renal fibrogenesis (Grande et al., 2015, LeBleu et al., 2013, Lovisa et al., 2015). Kidney TECs were isolated from wild-type (WT), *Ctsb*^*–/–*^, *Ctsl*^*–/–*^, *Ctss*^*–/–*^, and *Ctsk*^*–/–*^ mice and characterized by immunofluorescent staining for the epithelial marker E-cadherin and for the proximal tubule epithelial cell marker aquaporin-1 (Figure S1A). Over 95% of Cultured TECs displayed E-cadherin by flow cytometry analysis (Figure S1B). TGF-β promoted acquisition of a fibrotic phenotype by TECs characterized by reduced E-cadherin expression and increased α-SMA expression (Figure S1C). Immunoblot analysis (Figure 1A) and immunofluorescent staining (Figure 1B) showed that TGF-β increased the expression of CatS and CatK but reduced the expression of CatB and CatL in mouse kidney TECs. To test whether differential expression of these cathepsins affected differently TEC fibrotic phenotype changes or differentiation into myofibroblasts, we treated TECs from cathepsin-deficient mice with TGF-β. Both immunoblot analysis (Figure 1C) and immunofluorescent staining (Figure 1D) showed that deficiency of CatS or CatK reduced the production of α-SMA and fibronectin, whereas deficiency of CatB or CatL increased the expression of these fibrotic proteins in TECs after TGF-β stimulation, as compared with TECs from WT control mice. Therefore, in mouse TECs, TGF-β acted differently in regulating the expression of different cathepsins and different cathepsins also showed varying activities in regulating the expression of TGF-β-induced profibrotic genes.

### Differential Activity of Cathepsins in Kidney TEC Smad Activation

**Figure 2:**
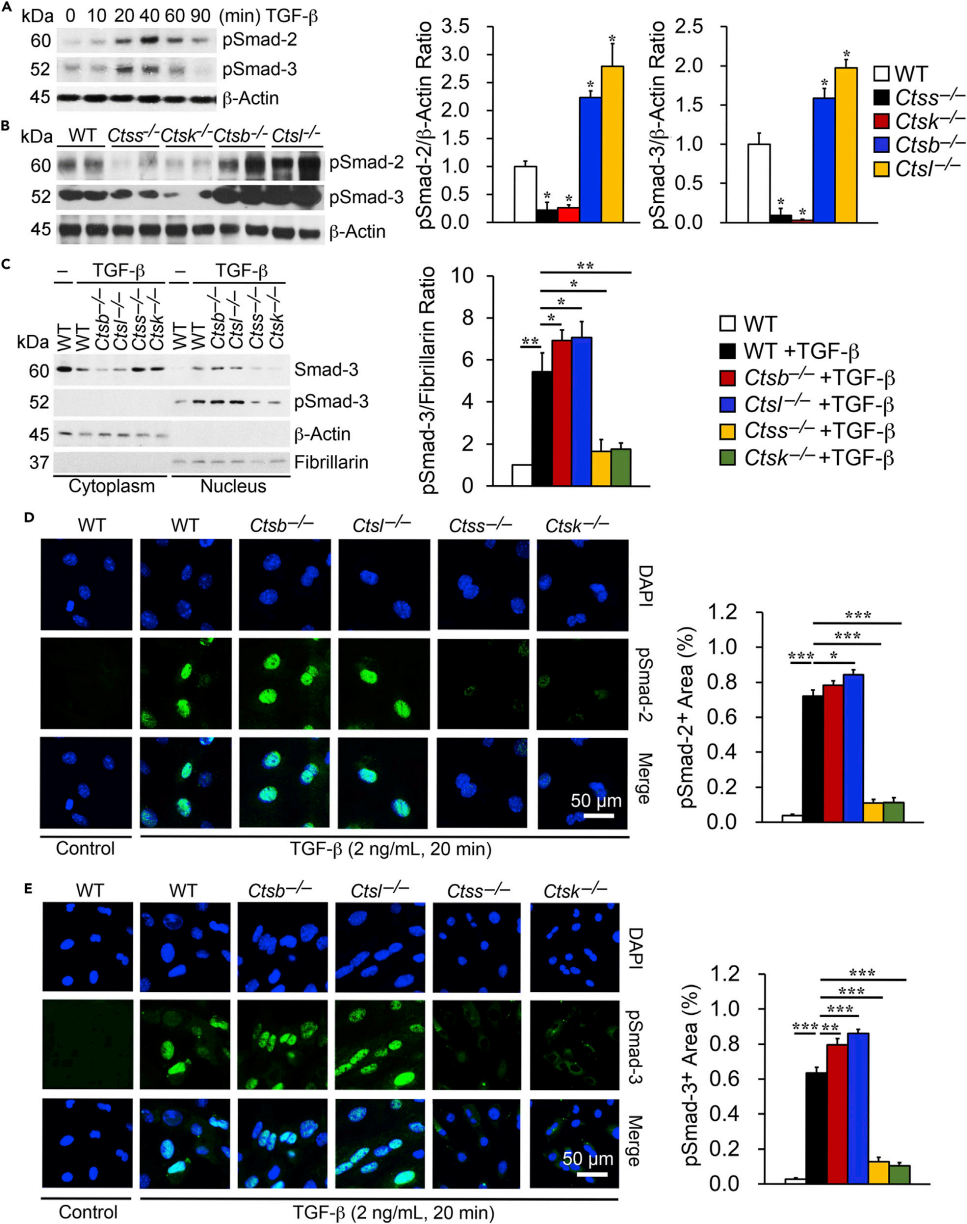
Cathepsin Activity Regulates TGF-β (2 ng/mL) Downstream Smad-2 and Smad-3 Activation in Mouse Kidney TECs (A) Immunoblot analysis of pSmad-2 and pSmad-3 in kidney TECs with and without TGF-β stimulation at different time points. (B) Immunoblot analysis of pSmad-2 and pSmad-3 and quantification relative to β-Actin in kidney TECs from WT, *Ctsb*^*−/−*^, *Ctsl*^*−/−*^, *Ctss*^*−/−*^, and *Ctsk*^*−/−*^ mice after TGF-β stimulation for 20 min. (C–E) (C) Immunoblot analysis of Smad-3 and pSmad-3 in the cytoplasm and nucleus and quantification of nuclear pSmad-3 relative to fibrillarin after TGF-β stimulation for 20 min in different kidney TECs. Immunofluorescence staining of pSmad-2 (D, green) and pSmad-3 (E, green) with nuclei counterstained with DAPI (blue) and quantification of positive areas. Scale bar: 50 μm. Data are representative of four independent experiments. ∗p < 0.05, ∗∗p < 0.01, ∗∗∗p < 0.001.

TGF-β signaling and Smad activation drive TEC myofibroblast differentiation. Translocation of pSmad-2/3 to the nucleus precedes profibrotic gene transcription and tissue fibrosis (Grande et al., 2015, Lan, 2003, Lovisa et al., 2015, Nikolic-Paterson et al., 2014). Altered profibrotic gene expression in TECs from different cathepsin-deficient mice suggests that these cathepsins exert different activities in Smad activation. Phosphorylation of Smad-2 and Smad-3 peaked at 20 to 40 min (Figure 2A) when TECs from WT mice were treated with TGF-β (2 ng/mL). Under the same conditions (2 ng/mL TGF-β, 20 min), pSmad-2/3 in TECs from CatS- and CatK-deficient mice showed greatly reduced levels, yet displayed highly increased levels in TECs from CatB- and CatL-deficient mice (Figure 2B). Increased pSmad-3 activation in TECs from CatB- and CatL-deficient mice and decreased pSmad-3 activation in TECs from CatS- and CatK-deficient mice occurred in the nuclear fraction, whereas total Smad-3 expression revealed itself in both nuclear and cytoplasmic fractions, as shown by immunoblot analysis. Fibrillarin and β-actin immunoblots validated the separation of nuclear and cytoplasmic fractions (Figure 2C). Consistent with the immunoblot results, after TGF-β stimulation, positive immunofluorescent staining with anti-pSmad-2 (Figure 2D) and anti-pSmad-3 (Figure 2E) antibodies detected increased nuclear pSmad-2/3 in TECs from *Ctsb*^*–/–*^ and *Ctsl*^*–/–*^ mice but reduced nuclear pSmad-2/3 in those from *Ctss*^*–/–*^ and *Ctsk*^*–/–*^ mice. These observations suggested that CatB and CatL inhibited pSmad-2/3 nuclear translocation but that CatS and CatK promoted pSmad-2/3 nuclear translocation.

### Cathepsin Activities in Nuclear Membrane Transporter Protein Expression and Immunocomplex Formation

**Figure 3:**
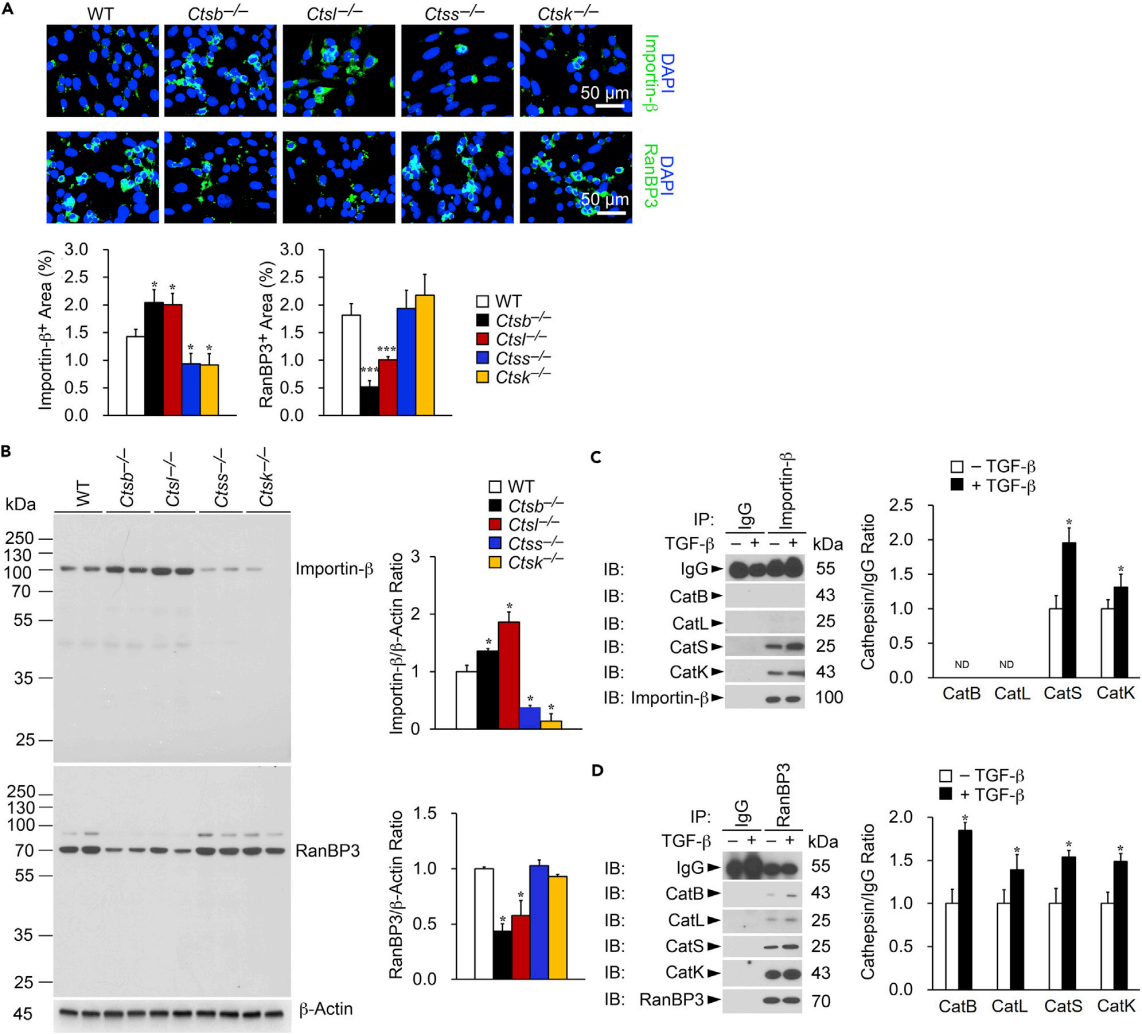
Differential Roles of Cathepsins in Expression and Immunocomplex Formation with Nuclei Membrane Transporters Importin-β and RanBP3 (A–D) Immunofluorescent staining of importin-β (green) and RanBP3 (green) with nuclei counterstained with DAPI (blue) and quantification of positive areas (A) and immunoblot of importin-β and RanBP3 and quantification relative to β-Actin (B) in TECs from different mice as indicated after TGF-β stimulation (2 ng/mL). Immunoprecipitation of anti-importin-β (C) and anti-RanBP-3 (D) of TEC lysates (250 μg), followed by immunoblot detection of different cathepsins and quantification relative to IgG isotype. Mouse IgG isotype (10 μg) was used for immunoprecipitation negative controls. Scale bar: 50 μm. Data are representative of four independent experiments. ∗p < 0.05, ∗∗p < 0.01, ∗∗∗p < 0.001.

Activated Smad-2/3 and common Smad-4 form a complex that possess intrinsic nucleocytoplasmic shuttling capacity to control its target gene transcriptional activities by changing its subcellular distribution (Feng and Derynck, 2005). Nuclear membrane transport factors mediate this process, such as importin-β and RanBP3, which recognize and bind directly to the complex to trigger its nuclear import and export translocations (Dai et al., 2009, Xiao et al., 2000). Results showed increased importin-β expression in TGF-β-activated TECs from *Ctsb*^*–/–*^ and *Ctsl*^*–/–*^ mice but decreased expression in TECs from *Ctss*^*–/–*^ and *Ctsk*^*–/–*^ mice via immunofluorescent staining and immunoblot analysis (Figures 3A and 3B). Changes in importin-β expression in TECs may affect directly pSmad-2/3 nuclear import (Xiao et al., 2000). In contrast, TECs from *Ctsb*^*–/–*^ and *Ctsl*^*–/–*^ mice showed reduced nuclear exporter RanBP3, although CatS- and CatK-deficiency in TECs did not affect RanBP3 expression (Figures 3A and 3B). These observations agree with the increased pSmad-2/3 in the nucleus of TECs from *Ctsb*^*–/–*^ and *Ctsl*^*–/–*^ mice and reduced nucleus pSmad-2/3 in TECs from *Ctss*^*–/–*^ and *Ctsk*^*–/–*^ mice (Figures 2B–2E). To test whether these cathepsins might interact with importin-β and RanBP3 differently, we performed immunoprecipitation with importin-β or RanBP3 antibodies, followed by immunoblotting with antibodies that recognize different cathepsins. CatS and CatK, but negligibly CatB and CatL, interacted with the nuclear membrane importer importin-β and formed immunocomplexes (Figure 3C). Similarly, CatS or CatK interacted with the nuclear exporter RanBP3 and formed immunocomplexes to a much greater extent than did CatB or CatL (Figure 3D). TGF-β increased the formation of these immunocomplexes (Figures 3C and 3D). These results suggest that CatS or CatK facilitated pSmad-2/3 nuclear-cytoplasmic shuttling more efficiently than did CatB or CatL. Immunofluorescent double staining co-localized these cathepsins to nuclear membrane importin-β and RanBP3 (Figure S2). Therefore, co-localization between cathepsins and importin-β and RanBP3 by immunofluorescent double staining does not necessarily mean their interaction and immunocomplex formation between cathepsins and these nuclear membrane proteins may not indicate their direct or indirect interactions.

### Cathepsin Activities in Plasma Membrane TGF-β Receptor Expression and Immunocomplex Formation

**Figure 4:**
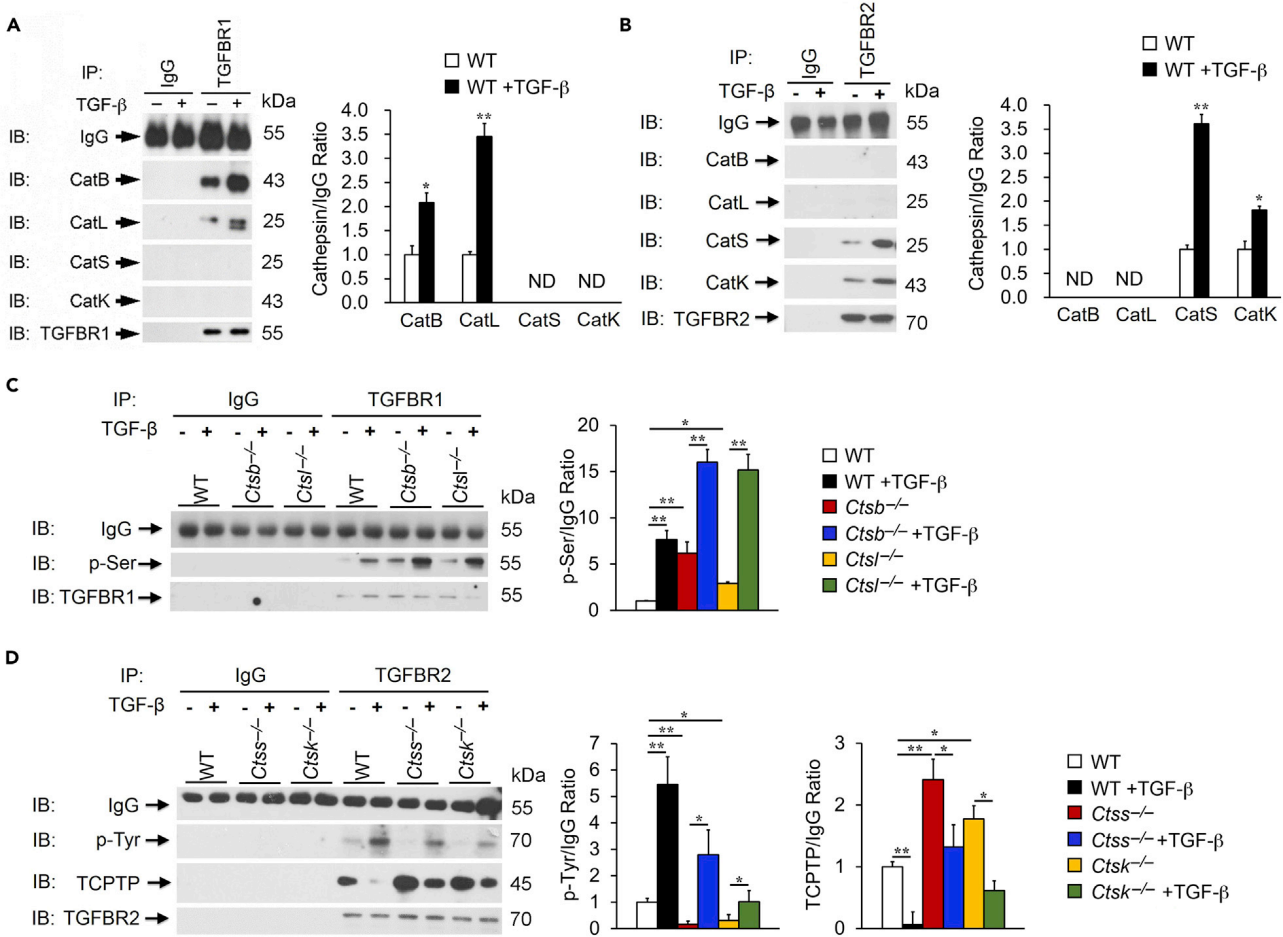
Differential Roles of Cathepsins in Immunocomplex Formation with and Activation of TGF-β Receptor-1 (TGFBR1) and TGF-β Receptor-2 (TGFBR2) (A and B) Immunoprecipitation of TGFBR1 (A) and TGFBR2 (B) of WT kidney TECs treated with or without TGF-β were followed by immunoblot analysis of different cathepsins as indicated and quantification relative to IgG. Goat IgG isotype was used as experimental negative control. (C) TGFBR1 immunoprecipitation followed by p-Ser and TGFBR1 immunoblot analysis to detect serine phosphorylation of TGFBR1 from TECs from WT, *Ctsb*^*−/−*^, and *Ctsl*^*−/−*^ mouse kidneys with and without TGF-β stimulation and quantification relative to IgG isotype. (D) TGFBR2 immunoprecipitation followed by p-Tyr, TGFBR1, and TCPTP immunoblot analyses to detect tyrosine phosphorylation of TGFBR1 and TGFBR2-TCPTP complex formation in TECs from WT, *Ctss*^*−/−*^, and *Ctsk*^*−/−*^ kidneys with and without TGF-β stimulation and quantification relative to IgG isotype. Data representative of four independent experiments. ∗p < 0.05, ∗∗p < 0.01.

Different activities of cathepsins in nuclear membrane importin-β, RanBP3 expression, and immunocomplex formation (Figure 3) may explain the differences of these cathepsins in Smad activation and ECM expression (Figure 2). We further tested whether these cathepsins also interact with TGF-β receptors differently, which may represent another layer of regulation of TGF-β signaling and Smad activation by cathepsins. TGF-β binds and activates TGFBR2, followed by recruiting and activating TGFBR1, essential for downstream Smad-dependent fibrotic signaling and tissue fibrosis (Massague, 1998, Roberts, 1998, Schnaper et al., 2002). In the absence of TGF-β, C-terminal tyrosine residues of TGFBR2 are dephosphorylated by T cell protein tyrosine phosphatase (TCPTP), resulting in TGFBR-dependent inhibition of fibrotic signaling (Chen et al., 2014). Therefore, TCPTP negatively regulates TGFBR2 (Allison, 2014). Immunoprecipitation of TEC lysate from WT mice with anti-TGFBR1 and anti-TGFBR2 antibodies followed by cathepsin immunoblot analysis demonstrated that CatB and CatL formed immunocomplexes with the TGFBR1, whereas CatS and CatK preferentially formed immunocomplexes with the TGFBR2. TGF-β enhanced the formation of these cathepsin-TGFBR immunocomplexes (Figures 4A and 4B). Immunofluorescent double staining colocalized these cathepsins to TGF-β receptors TGFBR1 and TGFBR2 in TECs after TGF-β activation (Figure S3), although the interactions between TGFBR1 and CatS or CatK and between TGFBR2 and CatB and CatL remained negligible by immunoblot analysis (Figures 4A and 4B). Consistent with enhanced interactions between TGFBR1 and CatB or CatL, TGF-β-induced serine phosphorylation on TGFBR1 increased in TECs from *Ctsb*^*–/–*^ and *Ctsl*^*–/–*^ mice, as determined by TGFBR1 immunoprecipitation followed by anti-p-serine (p-Ser) monoclonal antibody immunoblot analysis. TGFBR1 immunoblot helped ensure equal TGFBR1 immunoprecipitation (Figure 4C). These observations suggest a role of CatB and CatL in interacting with and inhibiting TGFBR1 activation. Absence of any one of them increased TGFBR1 serine phosphorylation. Double deficiency of both CatB and CatL may further increase TGFBR1 activation, although this study did not test this possibility. In contrast, it is also possible that the remaining CatB in TECs from *Ctsl*^*–/–*^ mice or the remaining CatL in TECs from *Ctsb*^*–/–*^ mice may bind and activate TGFBR1. Yet, increased TGFBR1 serine phosphorylation in TECs from *Ctsl*^*–/–*^ or *Ctsb*^*–/–*^ mice may not support this possibility. TGFBR2 activation in TECs was assessed by TGFBR2 immunoprecipitation of TEC lysates followed by immunoblot analysis of tyrosine phosphorylation (p-Tyr) using an anti-p-Tyr monoclonal antibody. TGF-β-induced p-Tyr on TGFBR2 decreased in TECs from *Ctss*^*–/–*^ and *Ctsk*^*–/–*^ mice, compared with TECs from WT control mice (Figure 4D), suggesting a role of CatS and CatK in interacting and activating TGFBR2. Consistent with this hypothesis, elevated interaction of TGFBR2 with its inhibitor TCPTP in TECs from *Ctss*^*–/–*^ and *Ctsk*^*–/–*^ mice before and after TGF-β induction was revealed by TGFBR2 immunoprecipitation followed by immunoblot analysis using the anti-TCPTP antibody. TGFBR2 immunoblot helped ensure equal TGFBR2 precipitation (Figure 4D).

### TEC Cathepsin Expression in UUO-Injured Mouse Kidneys

**Figure 5:**
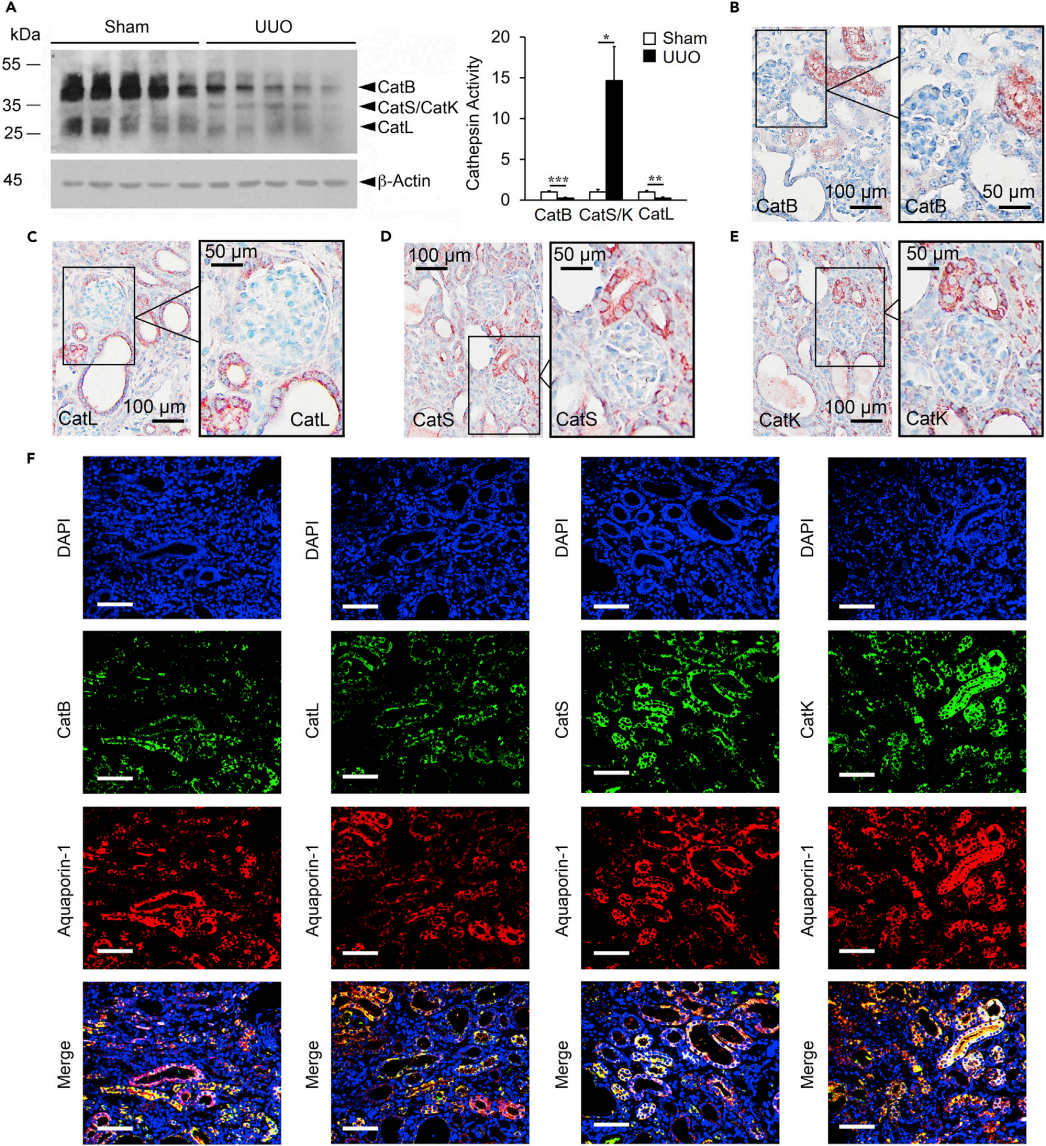
Cathepsin Expression and Localization to TECs in Kidneys from UUO-Injured WT Mice (A) Kidney tissue extract JPM labeling to detect active cathepsins and quantification relative to β-Actin at 14 days after sham or UUO injury. (B) Immunostaining of kidney sections to detect the expression of cathepsin B in UUO-treated kidneys. Scale: 100 μm; Inset scale: 50 µm. (C) Immunostaining of kidney sections to detect the expression of cathepsin L in UUO-treated kidneys. Scale: 100 μm; Inset scale: 50 µm. (D) Immunostaining of kidney sections to detect the expression of cathepsin S in UUO-treated kidneys. Scale: 100 μm; Inset scale: 50 µm. (E) Immunostaining of kidney sections to detect the expression of cathepsin K in UUO-treated kidneys. Scale: 100 μm; Inset scale: 50 µm. (F) Immunofluorescent double staining of cathepsins (green) and renal TEC marker aquaporin-1 (red), with nuclei counterstained with DAPI (blue). Scale bar: 100 μm. n = 8–10 per group. ∗p < 0.05, ∗∗p < 0.01, ∗∗∗p < 0.001.

Differential roles of CatS and CatK versus CatB and CatL in profibrotic protein production (Figure 1), Smad activation (Figure 2), interactions with nuclear membrane Smad transporter proteins (Figure 3), and interactions with plasma membrane TGF-β receptors (Figure 4) all suggest that these cathepsins contribute differently to tissue fibrosis. To test this conjecture in a fibrosis-dependent pathological process *in vivo* we studied obstructive uropathy in mice. UUO is used broadly to study kidney progressive proximal tubule injury and fibrosis and their underlying mechanisms (Bonventre, 2014, Humphreys et al., 2013, Li et al., 2016, Lovisa et al., 2015, Yang et al., 2010, Zhou et al., 2010). After 14 days, UUO caused extensive proximal tubular degeneration and apoptosis, as determined by loss of E-cadherin immunoreactivity and increase of TUNEL-positive cells (Figures S4A and S4B), which accompany collecting duct dilatation (Figure S4A) and interstitial fibrosis (Forbes et al., 2011, Forbes et al., 2012). Recent studies showed that CatK and CatS contributed to ischemia- or hypoxia-induced vascular cell apoptosis via the Notch1, PPAR-γ, HADC6, and MAPK pathways (Jiang et al., 2014, Li et al., 2015, Wu et al., 2016). Consistent with these hypotheses, we detected many fewer TUNEL-positive cells in the kidneys from *Ctss*^*–/–*^ and *Ctsk*^*–/–*^ mice than in those from WT mice after UUO injury (Figure S4B). Immunofluorescent staining also demonstrated the loss of proximal tubular aquaporin-1 expression and an increase of cleaved caspase-3-positive apoptotic cells (Figure S4C). Mouse kidneys after UUO-induced injury showed reduced CatB and CatL activities but increased CatS and CatK activities, as determined by cathepsin active site labeling with biotinylated-JPM (Chen et al., 2013, Sun et al., 2011, Sun et al., 2012) (Figure 5A). Immunostaining revealed the expression of these cathepsins in the proximal tubules in normal mouse kidneys (Figure S5). Immunostaining also localized these cathepsins mainly to the proximal tubules in UUO-injured kidneys (Figures 5B–5E). Some cells in the glomeruli were CatS- and CatK-positive and these cells may be inflammatory infiltrates, yet not defined (Figures 5D and 5E). Immunofluorescent double staining further localized cathepsins to aquaporin-1-positive proximal renal TECs (Jenq et al., 1999, Wang et al., 2015) in UUO-injured kidneys (Figure 5F). Although not tested in this study, inflammatory cell infiltration and expression of CatS and CatK in the glomeruli may also contribute to the kidney fibrosis after UUO injury or other pathological stimuli (Cheng et al., 2011) with different mechanisms from regulating the TEC TGF-β signaling pathway.

### Differential Roles of Cathepsins in UUO-Induced Kidney Fibrosis

**Figure 6:**
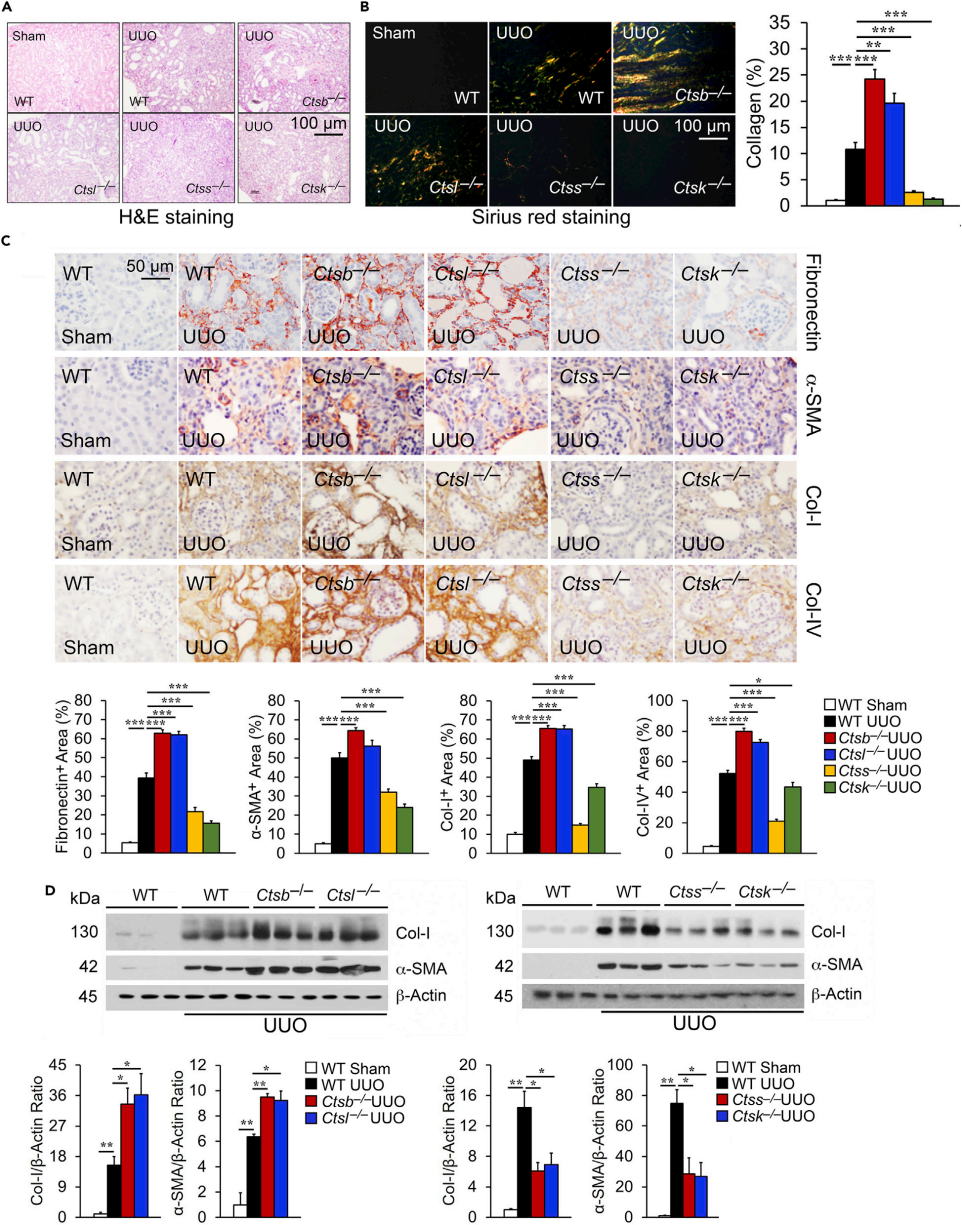
Differential Roles of Cathepsins in Mouse Post-UUO Renal Fibrosis (A–D) HandE (A) and Sirius red staining and quantification of positive areas (B) of kidneys from sham WT or 14 days post-UUO-treated WT, *Ctsb*^*−/−*^, *Ctsl*^*−/−*^*, Ctss*^*−/−*^, and *Ctsk*^*−/−*^ mice. Immunostaining (C) and immunoblots (D) detected matrix proteins fibronectin, α-SMA, collagen-I, and collagen-IV and quantification in kidneys from the same sets of mice. Scale bar in (A) and (D): 100 μm. Scale bar in (C): 50 μm. n = 8–10 per group. ∗p < 0.05, ∗∗p < 0.01, ∗∗∗p < 0.001.

**Figure 7:**
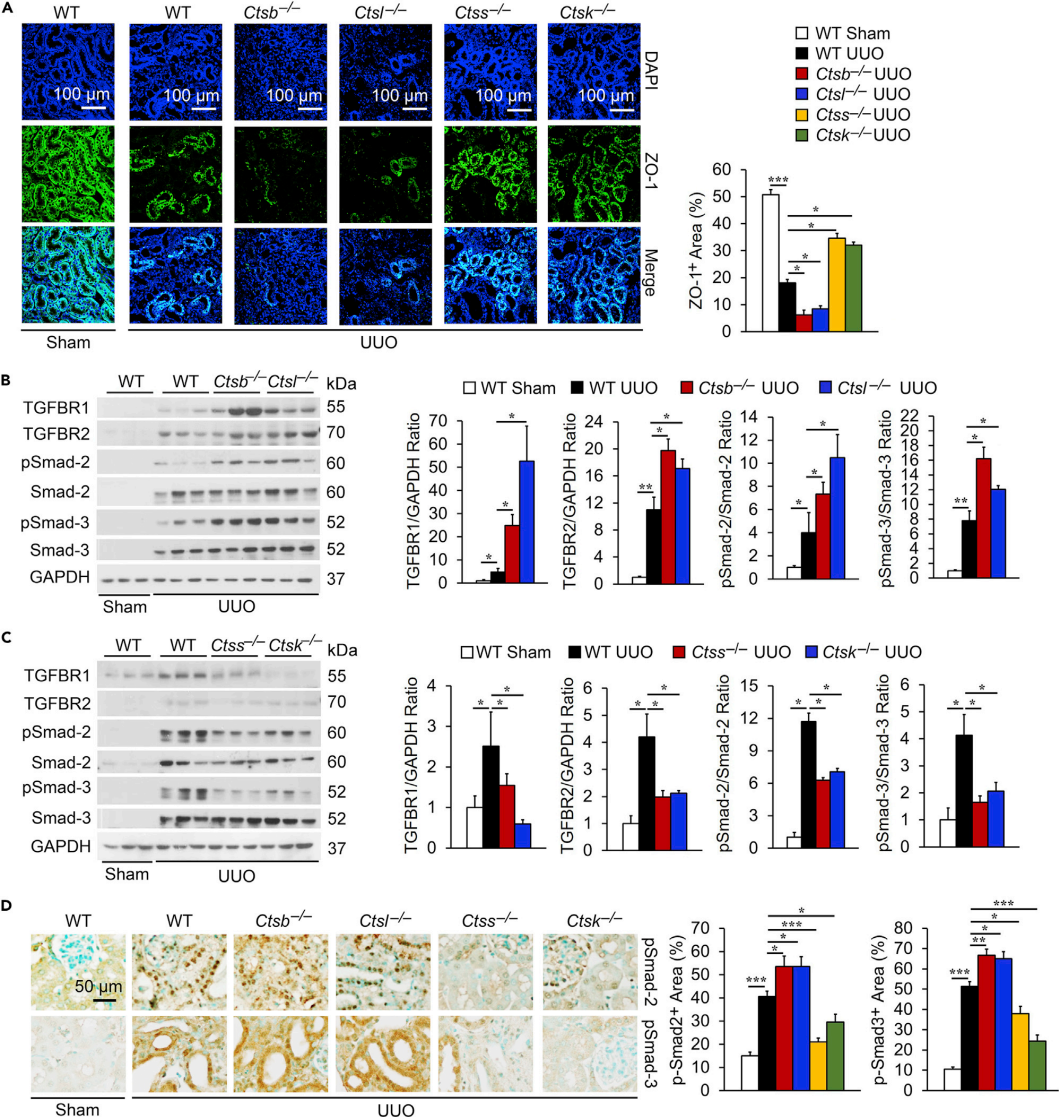
Differential Role of Cathepsins in UUO-Induced Kidney Fibrosis (A) Immunofluorescent staining of epithelial cell marker ZO-1 (green) with nuclei counterstained with DAPI (blue) in kidneys from sham WT or 14 days post-UUO WT, *Ctsb*^*−/−*^, *Ctsl*^*−/−*^*, Ctss*^*−/−*^, and *Ctsk*^*−/−*^ mice and quantification of positive areas. (B) Immunoblot analysis of TGFBR1, TGFBR2, pSmad-2, and pSmad-3 and quantification relative to GAPDH in kidney extracts from sham WT or 14 days post-UUO WT, *Ctsb*^*−/−*^, and *Ctsl*^*−/−*^ mice. (C) Immunoblot analysis of TGFBR1, TGFBR2, pSmad-2, and pSmad-3 and quantification relative to GAPDH in kidney extracts from sham WT or 14 days post-UUO WT, *Ctss*^*−/−*^, and *Ctsk*^*−/−*^ mice. (D) Immunostaining of pSmad-2 and pSmad-3 in kidneys from the same sets of mice and quantification of positive areas. Scale in A: 100 μm. Scale in D: 50 μm. n = 8–10 per group. ∗p < 0.05, ∗∗p < 0.01, ∗∗∗p < 0.001.

UUO-induced kidney injury leads to extensive interstitial fibrosis (Forbes et al., 2012). Reduced CatB or CatL activity but increased CatS or CatK activity in UUO-injured kidneys (Figure 5A) suggest their differential roles in post-UUO injury kidney fibrosis. H&E (Figure 6A) and Sirius red staining (Figure 6B) revealed that *Ctsb*^*–/–*^ and *Ctsl*^*–/–*^ mice developed greater tubular dilatation and collagen deposition than WT control mice, yet *Ctss*^*–/–*^ and *Ctsk*^*–/–*^ mice were protected from post-UUO tubular dilatation and collagen deposition. Immunostaining (Figure 6C) and immunoblot analyses (Figure 6D) supported these conclusions. UUO induced kidney production of the myofibroblast marker α-SMA (LeBleu et al., 2013) and ECM proteins fibronectin, collagen type-I (Col-I), and type-IV (Col-IV) in WT mice. Kidneys from *Ctsb*^*–/–*^ and *Ctsl*^*–/–*^ mice showed greatly enhanced accumulation of these ECM constituents, in contrast to those from *Ctss*^*–/–*^and *Ctsk*^*–/–*^ mice. Increased α-SMA expression in kidneys from UUO-treated mice suggests enhanced TEC-to-myofibroblast transition. Kidney zonula occludens-1 (ZO-1)-positive (Figure 7A) and E-cadherin-positive (Figure S6) TEC levels appeared reduced in kidneys from UUO-treated WT mice and further reduced in kidneys from *Ctsb*^*–/–*^ and *Ctsl*^*–/–*^ mice. In contrast, ZO-1- and E-cadherin-positive TECs in kidneys from UUO-treated *Ctss*^*–/–*^and *Ctsk*^*–/–*^ mice underwent greater preservation than those in WT mice (Figures 7A and S6), consistent with their reduced kidney α-SMA (Figures 6C and 6D). Kidneys from UUO-treated WT mice also showed increased TGF-β receptors TGFBR1 and TGFBR2, and pSmad-2/3. Such increases were further enhanced in kidneys from UUO-treated *Ctsb*^*–/–*^ and *Ctsl*^*–/–*^ mice but blunted in kidneys from *Ctss*^*–/–*^and *Ctsk*^*–/–*^ mice, as determined by both immunoblot analysis and immunostaining (Figures 7B–7D).

## Discussion

This study presents differential activities of prominent members of the cathepsin family in interacting with the plasma membrane TGF-β receptors and nuclear membrane transporters. Both properties may contribute to their differential activities in Smad activation, ECM production, and kidney fibrosis from cultured mouse kidney TECs and UUO-induced kidney injury in mice. On the cell membrane, TGF-β interacts with and activates TGFBR2. Activated TGFBR2 mediates TGFBR1 activation. During this process, TCPTP dephosphorylates the p-tyrosine residues located on the C-terminal intracellular tail of the TGFBR2 and acts as a TGFBR2 negative regulator (Chen et al., 2014). Therefore, any molecule that interacts with TGFBR2, TGFBR1, or TCPTP may affect the TGF-β signaling cascade.

This study showed that CatS or CatK formed immunocomplexes with TGFBR2 (Figure 4B). Although immunocomplex formation does not prove direct interaction between cathepsins and TGFBR2, reduced downstream Smad activation (Figures 2B, 2D, and 2E) in TECs from *Ctss*^*–/–*^ and *Ctsk*^*–/–*^ mice and reduced kidney ECM production from these mice support the functional consequences of this interaction (Figures 6B–6D). Current evidence does not explain why the interaction between CatS and CatK and TGFBR2 enhanced the downstream TGF-β signaling. Several possibilities may remain. As proteases, CatS and CatK may bind to TGFBR2 and mediate TGFBR2 proteolytic activation. Yet, immunoblot analysis of kidney extracts from *Ctss*^*–/–*^and *Ctsk*^*–/–*^ mice did not detect accumulation of any fragments higher than the predicted 70-kDa TGFBR2 (data not shown), although it is still possible that the monoclonal antibody that recognizes the Ile24 to Asp184 fragment of TGFBR2 may have weak immunoreactivity to the unprocessed TGFBR2 precursors. CatS and CatK may also compete the binding of TGFBR2 with TCPTP. The absence of CatS or CatK gave more chances for TCPTP to attack and suppress TGFRB2 activity. Our data in Figure 4D supported this possibility. In the presence or absence of TGF-β, we detected much more TGFBR2-bound TCPTP in TECs from *Ctss*^*–/–*^ and *Ctsk*^*–/–*^ mice than those from WT mice. CatS and CatK expression may also enhance the production of TGFBR2 at the transcriptional or translational levels. Data presented in Figure 7C may support this possibility. Kidneys from *Ctss*^*–/–*^and *Ctsk*^*–/–*^ mice showed reduction of TGFBR2. Yet, the magnitude of reductions of downstream ECM production (Figure 1D) and nuclear pSmad-2/3 translocation (Figures 2B–2E) in TECs from *Ctss*^*–/–*^and *Ctsk*^*–/–*^ mice support the hypothesis that CatS and CatK compete the interaction of TGFBR2 with TCPTP. Therefore, TECs from *Ctss*^*–/–*^and *Ctsk*^*–/–*^ mice showed increased TCPTP binding on TGFBR2 and reduced p-Tyr of TGFBR2 (Figure 4D). In contrast, CatB and CatL acted differently from CatS and CatK. The interaction between CatB and CatL and TGFBR1 (Figure 4A) reduced TGFBR1 activity (p-Ser) (Figure 4C). These data suggest that CatB and CatL negatively regulate TGFBR1. Plasma membrane CatB and CatL may mediate TGFBR1 degradation, which could explain the increased TGFBR1 in kidney extracts from *Ctsb*^*–/–*^ and *Ctsl*^*–/–*^ mice (Figure 7B). Alternatively, the formation of immunocomplexes between CatB and CatL and TGFBR1 may prevent the interaction between TGFBR1 and TGFBR2, another site of interference with downstream signaling. The absence of CatB and CatL may facilitate the interaction between the two receptors on the plasma membrane.

On the nuclear membrane, importin-β mediates pSmad-3 translocation from the cytoplasm to the nucleus by binding to pSmad-3 (Xiao et al., 2000). In contrast, RanBP3 exports Smad-2/3 complex also by binding to the Smad-2/3 complex after dephosphorylation by a serine/threonine phosphatase PPM1A as a mechanism to terminate Smad signaling (Dai et al., 2009). Therefore, any interference of importin-β and RanBP3, including expressional changes of these nuclear membrane transporters, may affect their binding with pSmad-2/3 or Smad-2/3, thereby changing the translocation efficiency of Smad complexes. This study showed that CatS and CatK promoted importin-β expression but that CatB and CatL inhibited importin-β expression (Figures 3A and 3B top panel). These expression profile changes may explain enhanced nuclear pSmad-2/3 in TECs from *Ctsb*^*–/–*^ and *Ctsl*^*–/–*^ mice and suppressed nuclear pSmad-2/3 in TECs from *Ctss*^*–/–*^and *Ctsk*^*–/–*^ mice (Figures 2B–2E). In contrast to importin-β, RanBP3 showed a reduced expression in TECs from *Ctsb*^*–/–*^ and *Ctsl*^*–/–*^ mice but not in those from *Ctss*^*–/–*^and *Ctsk*^*–/–*^ mice (Figure 3B bottom panel), suggesting that only CatB and CatL increased RanBP3 expression, which may increase Smad-2/3 export from the nucleus to cytoplasm, thereby reducing nuclear pSmad-2/3 activity.

This study showed that cathepsin activities regulate the expression of importin-β, RanBP3, TGFBR1, and TGFBR2 (Figure 3A, 3B, 7B, and 7C). How cathepsins controlled the expression of these genes remains unexplained. This situation resembles that we encountered when we found that CatS-deficiency affected the expression of α-SMA (Chen et al., 2013) and CatK- or CatL-deficiency affected the expression of other proteases (Chen et al., 2013, Sun et al., 2011, Sun et al., 2012). We now can explain that CatS-deficiency reduced α-SMA expression by controlling the expression and activities of importin-β, RanBP3, TGFBR1, and TGFBR2. Therefore, cathepsins may also control the transcriptional or translational machineries for other proteases (Sun et al., 2011, Sun et al., 2012).

In addition to enhance importin-β expression, CatS and CatK also preferred to interact or form immunocomplexes with importin-β (Figure 3C) and RanBP3 (Figure 3D) than did CatB and CatL, although CatB and CatL also colocalized to importin-β and RanBP3 by immunofluorescent double staining (Figure S2). Formation of immunocomplexes of CatS and CatK with importin-β may enhance the binding of pSmad-2/3 (Xiao et al., 2000) or increase the translocation efficiency of importin-β. The formation of immunocomplexes of CatS and CatK with RanBP3 may also affect the rate or efficiency of RanBP3 in nuclear protein export. As proteases, CatS and CatK interaction with RanBP3 may affect RanBP3 proteolytic processing or degradation. Yet, no evidence currently exists to support this notion. Immunoblot analysis of RanBP3 in TECs from *Ctss*^*–/–*^ and *Ctsk*^*–/–*^ mice did not detect accumulation of the 70-kDa RanBP3 or its precursors (Figure 3B). Together, our data did not support a major role of CatS and CatK or CatB and CatL in the proteolysis of importin-β or RanBP3, but rather formed immunocomplexes with or affected the expression of these nuclear membrane transporters, thereby facilitating pSmad-2/3 translocation by CatS and CatK or reducing pSmad-2/3 translocation by CatB and CatL. Of note, CatS- and CatK-induced TGF-β signaling may in turn increase the expression of CatS and CatK. CatB- and CatL-reduced TGF-β signaling may also reduce the expression of CatB and CatL (Figures 1A and 1B). Such reciprocal regulation occurred in mouse TECs. Future studies will explore further how and why TGF-β controlled differently the expression of CatS and CatK versus CatB and CatL.

The influences of cathepsins on tissue fibrosis may vary depending on the type of cathepsins, disease states, and cell types. In contrast to the present results regarding kidney fibrosis and TECs, CatK expression reduces lung and cardiac fibrosis. CatK deficiency exacerbated lung fibrosis, myocardial fibrosis, and cardiomyopathy (Buhling et al., 2004, Petermann et al., 2006, Zhang et al., 2011b). CatK overexpression limited these findings (Srivastava et al., 2008, Tang et al., 2009, Zhang et al., 2011a). TGF-β reduced CatK expression in fibroblasts (van den Brule et al., 2005) and CatL expression in lung epithelial cells (Chen et al., 2013). In contrast, CatB expression seems to promote lung and liver fibrosis. In human lung fibroblasts, CatB inhibition delayed fibroblast differentiation, decreased pSmad-2/3, and diminished α-SMA expression (Kasabova et al., 2014). CatB inhibition reduced CCl_4_-induced liver fibrosis (Moles et al., 2009, Moles et al., 2012). In our recent study of diet-induced nonalcoholic fatty liver disease (Wang et al., 2016), we found that CatB-deficiency reduced liver fibrosis (G.-P.S., Unpublished Data). Therefore, cathepsin activities in TGF-β signaling and tissue fibrosis may have disease-type- and cell-type-dependent properties.

Together, this study established differential roles of cathepsins in TGF-β signaling and renal fibrosis. The study also suggests that, instead of targeting cathepsins, mitigating tissue fibrosis by targeting TGF-β or its receptors may prove safer (Hill et al., 2001, Moon et al., 2006, Petersen et al., 2008, Russo et al., 2007), such as anti-TGF-β neutralizing antibody, endoglin (a TGF-β binding protein) antibody, soluble TGF-β type-II receptor, TGF-β antisense oligonucleotide, specific inhibitors of TGF-β receptor kinases (GW788388 and IN-1130), and pirfenidone (a small molecule TGF-β inhibitor), which recently received approval for the treatment of IPF (Carter, 2011). The use of cathepsin inhibitors to treat tissue fibrosis-associated diseases requires considerable caution, as a cathepsin that mitigates fibrosis of a given organ may aggravate fibrosis in other organs. Thus, these results shed mechanistic insight into how these closely related proteinases can exert opposing effects and how they can do so independent of their proteolytic properties. These findings have implications for understanding the regulation of tissue fibrosis in general as well as increasing our knowledge of cysteinyl proteinase biology.

### Limitations of the Study

This study reports that cysteinyl proteases cathepsins regulate TGF-β signaling and tissue fibrosis independent of their proteolytic activities. With different activities in regulating the expression of and in binding to the nuclear membrane Smad complex transporters importin-β and RanBP3 and plasma membrane TGF-β receptors TGFBR1 and TGFBR2, CatB and CatL exert opposite activities to those of CatS and CatK in controlling TGF-β signaling and tissue fibrosis. Several study limitations remain. First, we currently do not know how these cathepsins interact with importin-β, RanBP3, TGFBR1, and TGFBR2. This study only shows the immunocomplex formation between cathepsins and those nuclear and plasma membrane proteins. Such interaction may involve direct contact.

Yet, the identity of the specific primary residues responsible for the interaction on cathepsins and membrane proteins remains untested. Alternatively, such interaction can be indirect and involve other untested molecules. This study does not analyze the components from different cathepsin-membrane protein immunocomplexes. Second, this study uses kidney TECs and UUO-induced kidney injury model to study cathepsin activities in tissue fibrosis. It is possible that the observed activities of these cathepsins in kidney TECs may differ from those in other cell types or organs. This study does not test this hypothesis. Third, earlier studies show that deficiency of one cathepsin affects the expression of other cathepsins or matrix metalloproteinases in vascular smooth muscle cells, endothelial cells, and adipocytes (Sun et al., 2011, Sun et al., 2012, Yang et al., 2008). Yet, the underline mechanisms by which cathepsins regulate the expression of other proteases remain unknown because cathepsins have never been implicated in gene expression regulation besides their proteolytic function. This study offers an example that CatB, CatL, CatS, and CatK contribute to tissue fibrosis by regulating TGF-β signaling and ECM protein expression. This activity of cathepsins involves not only cathepsin immunocomplex formation with importin-β, RanBP3, TGFBR1, and TGFBR2, but also expression regulation of these nuclear and plasma membrane proteins. Yet, how these cathepsins regulate the expression of these membrane proteins remains unresolved.

## Methods

All methods can be found in the accompanying Transparent Methods supplemental file.

## Supplemental Information

Document S1. Transparent Methods and Figures S1–S6
