# Supplementary material for: Differential Roles of Cysteinyl Cathepsins in TGF-β Signaling and Tissue Fibrosis
Source: iScience. 2019 Aug 9;19:607–22. doi: 10.1016/j.isci.2019.08.014 (PMC6715892; doi:10.1016/j.isci.2019.08.014)
Supplement: Document S1. Transparent Methods and Figures S1–S6 [file mmc1.pdf]

ISCI, Volume *19*

Supplemental Information

Differential Roles of Cysteinyl Cathepsins in TGF-β Signaling and Tissue Fibrosis

Xian Zhang, Yi Zhou, Xueqing Yu, Qin Huang, Wenqian Fang, Jie Li, Joseph V. Bonventre, Galina K. Sukhova, Peter Libby, and Guo-Ping Shi

Supplemental Materials

Differential roles of cysteinyl cathepsins in TGF-β signaling and renal fibrosis

Xian Zhang, Yi Zhou, Xueqing Yu, Qin Huang, Wenqian Fang, Jie Li, Joseph V. Bonventre, Galina K. Sukhova, Peter Libby, Guo-Ping Shi

TRANSPARENT METHODS

Mice

Eight-week-old male *Ctsb*$^{-/-}$ (C57BL/6, N>7), *Ctsl*$^{-/-}$ mice (C57BL/6, N>7), *Cats*$^{-/-}$ (C57BL/6, N>10), *Ctsk*$^{-/-}$ (C57BL/6, N>9) and male wild type (C57BL/6, Jackson Laboratory, Bar Harbor, ME) were kept under pathogen-free conditions and housed in a temperature controlled room with a 12-hour light/dark cycle with ad libitum access to food and water. All animal procedures conformed to the Guide for the Care and Use of Laboratory Animals published by the US National Institutes of Health and were approved by the Harvard Medical School Standing Committee on Animals (protocol #03759).
To produce unilateral ureteral obstruction (UUO)-induced kidney fibrosis, we grouped mice randomly into sham or UUO groups. Surgery was performed by exposing the left ureter through a flank incision and then immediately closing the incision (sham) or double-ligating the left ureter with silk sutures (UUO). A minimum of 8~10 mice were used in each experimental group. All mice were sacrificed at 14 days after UUO.

Kidney histology analysis

Mouse kidney tissues were fixed with 4% paraformaldehyde (#P6148, Sigma-Aldrich, St. Louis, MO). Paraffin-embedded 4-μm kidney sections were prepared and stained with Masson's trichrome (#87019, Thermo Fisher Scientific, Waltham, MA), picrosirius red (#24901, Polysciences, Warrington, PA), apoptotic cell detection kit (#S7100, Millipore, Burlington, MA), and hematoxylin and eosin (H&E) (Sigma-Aldrich) according to the instructions. Five random fields/section were examined, and collagen content quantified using Image-Pro Plus.

For immunohistochemical analysis, the kidney sections were deparaffinized, rehydrated and incubated with the following primary antibodies: rabbit anti-cathepsin B (1:50, #PC41, Calbiochem, Billerica, MA), rabbit anti-cathepsin L (1:20, #168-10557, Raybiotech, Norcross, GA), rabbit anti-cathepsin S (was produced by our own group; 1:200), rabbit anti-cathepsin K (1:50, #PB9856, Boster, Pleasanton, CA), mouse anti-fibronectin (1:100, #ab6328, Abcam, Cambridge, MA), rabbit anti-α-SMA (1:750, #A2547, Sigma-Aldrich), rabbit anti-collagen-I (1:100, #ab292, Abcam) and rabbit anti-collagen-IV (1:100, #ab19808, Abcam), rabbit anti-pSmad-2 (1:30, #3108S, Cell Signaling Technology, Danvers, MA), rabbit anti-pSmad-3 (1:30, #9520S, Cell Signaling Technology) and anti-aquaporin-1 (1:100, #sc-9878, Santa Cruz Biotechnology, Dallas, TX), followed by appropriate biotin-conjugated secondary antibodies (1:500, Vector Laboratories, Burlingame, CA) and HRP-streptavidin (#P039701-2, DAKO, Carpinteria, CA). After detection with AEC chromogenic agent (#K3464, DAKO), slides were counterstained with hematoxylin (Sigma-Aldrich). Representative images were acquired with a Leica DM 1000 LED microscope.

Immunofluorescence staining of the kidney was performed using an established procedure (Zhou et al., 2017). Briefly, the 4-μm paraffin-embedded tissue sections were deparaffinized, rehydrated and incubated with the following primary antibodies at 4°C overnight: rabbit anti-cathepsin B (1:50, #PC41, Calbiochem), rabbit anti-cathepsin L (1:20, #168-10557, Raybiotech), rabbit anti-cathepsin S (from our own group; 1:100), rabbit anti-cathepsin K (1:50, #PB9856, Boster), rat anti-aquaporin-1 (1:50, #sc-9878, Santa Cruz Biotechnology), rat anti-ZO-1 (1:100, #sc-33725, Santa Cruz Biotechnology) and rat Alexa Fluor 594 anti-mouse E-cadherin (1:100, #147306, BioLegend, San Diego, CA), rabbit Alexa Fluor 594 anti-mouse cleaved caspase 3 (1:100, #8172, Cell Signaling Technology), followed by Alex Fluor 555 or 488-labelled secondary antibody detection. DAPI (#D9542, Sigma-Aldrich) was used to stain the nuclei. All images were collected by Olymbus Fluoview FV1000 confocal laser scanning microscopy.

For cell immunofluorescent analysis, cells were seeded onto 8-well chamber slides. After stimulation with or without TGF-β (BD Bioscience, San Jose, CA), the slide was fixed in cold methanol and immunostained with rabbit anti-cathepsin B (1:50, #PC41, Calbiochem), rabbit anti-cathepsin L (1:20, #168-10557, Raybiotech), rabbit anti-cathepsin S (1:100) or rabbit anti-cathepsin K (1:50, #PB9856, Boster), mouse anti-fibronectin (1:100, #ab6328, Abcam), rabbit anti-α-SMA (1:750, #A2547, Sigma-Aldrich), rabbit anti-pSmad-2 (1:30, #3108S, Cell Signaling Technology) and rabbit anti-pSmad-3 (1:30, #9520S, Cell Signaling Technology), rabbit anti-importin β (1:100, #8673S, Cell Signaling Technology), RanbP3-FITC (1:100, #NBP2-42672F, Novus Biologicals, Littleton, CO), rat anti-TGFBR-1 (1:100, #MAB5871, R&D Systems, Minneapolis, MN), and rat anti-TGFBR2 (1:100, #MAB532, R&D Systems) antibodies, rat anti-aquaporin-1 (1:50, #sc-9878, Santa Cruz Biotechnology), and rat Alexa Fluor 594 anti-mouse E-cadherin (1:100, #147306, BioLegend), followed by Alex Fluor 555 or 488-labeled secondary antibody detection (Thermo Fisher Scientific). Sections were analyzed with a confocal microscopy for subcellular localization of Alex Fluor 555 or 488 (Olymbus Fluoview FV1000; Olymbus).

Western blot analysis, JPM labeling, and immunoprecipitation
For immunoblot analysis, an equal amount of proteins extracted from kidney cortex or primary cells were separated on SDS-PAGEs, blotted, and detected with different antibodies, including mouse anti-α-SMA (1:1000, #A2547, Sigma-Aldrich), rabbit anti-collagen-I (1:1000, #ab292, Abcam), rat anti-TGFBR1 (1:1000, #MAB5871, R&D Systems), rat anti-TGFBR2 (1:1000, #MAB532, R&D Systems), mouse anti-Imp-β monoclonal antibody (1:1000, #MA3-070, Thermo Fisher Scientific), mouse anti-RanBP3 monoclonal antibody (1:1000, #sc-373678, Santa Cruz Biotechnology), rabbit anti-pSmad-2 (1:1000, #3108S, Cell Signaling Technology), rabbit anti-Smad-2 (1:1000, #5339S, Cell Signaling Technology), rabbit anti-pSmad-3 (1:1000, #9520S, Cell Signaling Technology), rabbit anti-Smad-3 (1:1000, #9523S, Cell signaling Technology), rabbit anti-cathepsin B (1:1000, # PC41, Calbiochem), rabbit anti-cathepsin L (1:1000, #168-10557, Raybiotech), rabbit anti-cathepsin S (1:3000, was produced by our own group), rabbit anti-cathepsin K (1:1000, #PB9856, Boster), rabbit anti-β-Actin (1:1000, #4970S, Cell Signaling Technology) and rabbit anti-GAPDH (1:100, #2118S, Cell Signaling Technology) antibodies. Cytoplasm and nucleus from kidney epithelial cells were extracted according to the manufacturer’s instructions (#78833, Thermo Fisher Scientific). β-Actin and fibrillarin (1:1000, #sc-166001, Santa Cruz Biotechnology) were used to reference protein in cytoplasm and nucleus, respectively.

JPM probe labeling was used to detect active cathepsins in kidney tissue extracts. Tissues or cells were lysed in a lysis buffer (pH 5.5) containing 1% Triton X-100, 40 mM sodium acetate, and 1 mM EDTA. Cathepsin active site JPM probe labeling was performed as described previously (Chen et al., 2013; Sun et al., 2012).

For immunoprecipitation, kidney epithelial cells were starved overnight in DMEM/F12 with 1% penicillin/streptomycin, followed by TGF-β stimulation for 24 h. Cells were lysed in an immunoprecipitation lysis buffer (0.025 M Tris, 0.15 M NaCl, 0.001 M EDTA, 1% NP-40, 5% glycerol; pH 7.4) and pre-cleared for 1 hour. Equal amounts of cell lysates (0.25 mg) were subsequently incubated overnight at 4°C with either antibody (goat anti-TGFBR1 antibody (10 µg, #AF587, R&D Systems), goat anti-TGFBR2 antibody (10 µg, # PA5-47719, Thermo Fisher Scientific), mouse anti-Imp-β monoclonal antibody (10 µg, #MA3-070, Thermo Fisher Scientific), mouse anti- RanbP3 monoclonal antibody (10 µg, #sc-373678, Santa Cruz Biotechnology) or IgG isotype control antibody (goat IgG isotype control antibody (10 µg, #02-6202, Thermo Fisher Scientific) or mouse IgG isotype control antibody (10 µg, #026502, Thermo Fisher Scientific)). The antibody-antigen complexes were captured, washed and eluted according to the manufacturer's instructions (#26149, Thermo Fisher Scientific). Immunoprecipitates were then resolved in SDS under reducing conditions, and followed by immunoblotting with rabbit anti-cathepsin B (1:1000, #PC41, Calbiochem), rabbit anti-cathepsin L (1:1000, #168-10557, Raybiotech), rabbit anti-cathepsin S (1:3000) or rabbit anti-cathepsin K (1:1000, #PB9856, Boster) antibodies to detect the immunocomplexes.

Isolation and culture of mouse kidney tubular epithelial cells

Primary tubular epithelial cells were prepared from renal cortical tissue from 8 to 10 weeks old male mice as previously described (Kimura et al., 2011). Briefly, mice were anesthetized and kidneys were harvested after cardial perfusion with PBS. Kidney cortex was separated, minced and incubated with 1 mg/ml collagenase type II (#LS004177, Worthington Biochemical Corp., Lakewood, NJ) at 37 °C for 20 min, followed by 1 G sedimentation for 1 minute to discard pellet. Cells were centrifuged for 5 min at 1000 g and washed twice with PBS before plating. Cells were cultured in DMEM/F12 supplemented with 10% FBS and 10 ng/ml murine EGF (#SRP3196, Sigma-Aldrich) and plated on 0.2% matrigel (1:500 diluted in PBS, #CB 40234, Thermo Fisher Scientific) coated plates. After starved with DMEM/F 12 with 1% penicillin/streptomycin overnight, cells were stimulated with or without 2 ng/ml TGF-β in serum free medium for the indicated time and lysed for western blot, immunoprecipitation, or fixed for immunofluorescent staining.

Flow cytometry

Primary kidney TECs were resuspended in PBS, and incubated with cell-surface marker PE anti-mouse E-cadherin antibody (1.0 µg per million cells in 100 µl, #147304, BioLegend) or isotype control for 30 min on ice, following separated by using a flow cytometer BD FACSCanto™ II (BD Biosciences). Data were analyzed using FlowJo V10.

Statistical analysis

All data were presented as means ± SEM. Because of relatively small sample sizes and often skewed data distribution, we selected the non-parametric Mann-Whitney *U* test for paired data sets and one-way ANOVA with post-hoc Bonferroni test was used for comparison among three or more groups to examine statistical significance. $P<0.05$ was considered as statistically significant.

REFERENCES

Chen, H., Wang, J., Xiang, M.X., Lin, Y., He, A., Jin, C.N., Guan, J., Sukhova, G.K., Libby, P., Wang, J.A., *et al.* (2013). Cathepsin S-mediated fibroblast trans-differentiation contributes to left ventricular remodelling after myocardial infarction. Cardiovasc Res *100*, 84-94.

Kimura, T., Takabatake, Y., Takahashi, A., Kaimori, J.Y., Matsui, I., Namba, T., Kitamura, H., Niimura, F., Matsusaka, T., Soga, T., *et al.* (2011). Autophagy protects the proximal tubule from degeneration and acute ischemic injury. J Am Soc Nephrol *22*, 902-913.

Sun, J., Sukhova, G.K., Zhang, J., Chen, H., Sjoberg, S., Libby, P., Xia, M., Xiong, N., Gelb, B.D., and Shi, G.P. (2012). Cathepsin K deficiency reduces elastase perfusion-induced abdominal aortic aneurysms in mice. Arterioscler Thromb Vasc Biol *32*, 15-23.

Zhou, Y., Chen, H., Liu, L., Yu, X., Sukhova, G.K., Yang, M., Zhang, L., Kyttaris, V.C., Tsokos, G.C., Stillman, I.E., *et al.* (2017). CD74 Deficiency Mitigates Systemic Lupus Erythematosus-like Autoimmunity and Pathological Findings in Mice. J Immunol *198*, 2568-2577.

SUPPLEMENTAL FIGURES

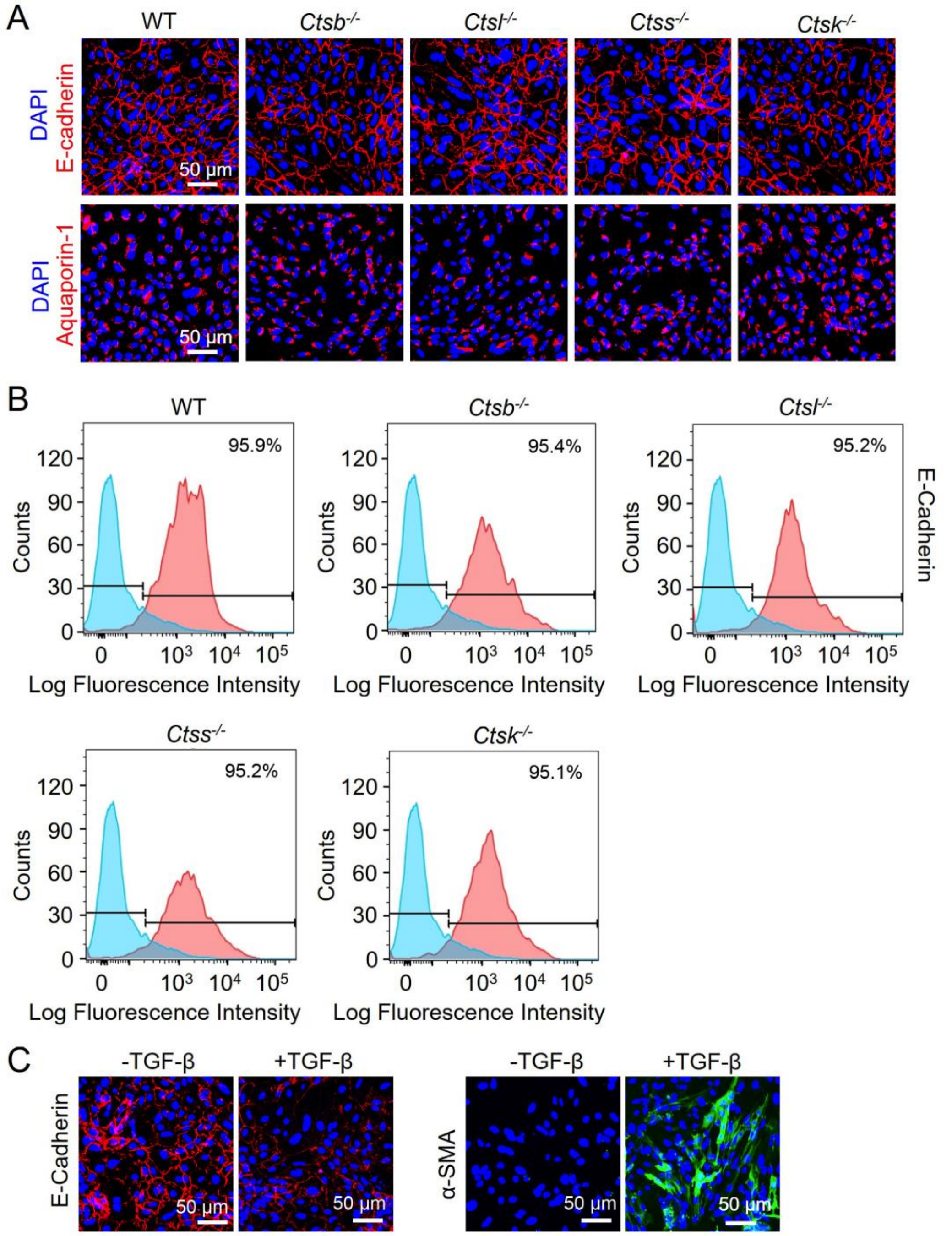

Figure S1. Characterization of renal cortical TECs isolated from wild-type, related to Figure 1. *Ctsb*$^{-/-}$, *Ctsl*$^{-/-}$, *Ctss*$^{-/-}$ and *Ctsk*$^{-/-}$ mice. **(A)** Immunofluorescent staining detected the expression of E-cadherin (red) or aquaporin-1 (red) with nuclei counterstained with DAPI (blue). **(B)** FACS analysis of TECs after staining with E-cadherin-PE antibody (red histogram) or rat IgG isotype control (blue histogram). **(C)** Immunofluorescent staining detected the expression of E-cadherin (red) or α-SMA (green) with nuclei counterstained with DAPI (blue) in WT mouse TECs treated without and with TGF-β (2 ng/mL, 24 hrs). Scale bar: 50 μm.

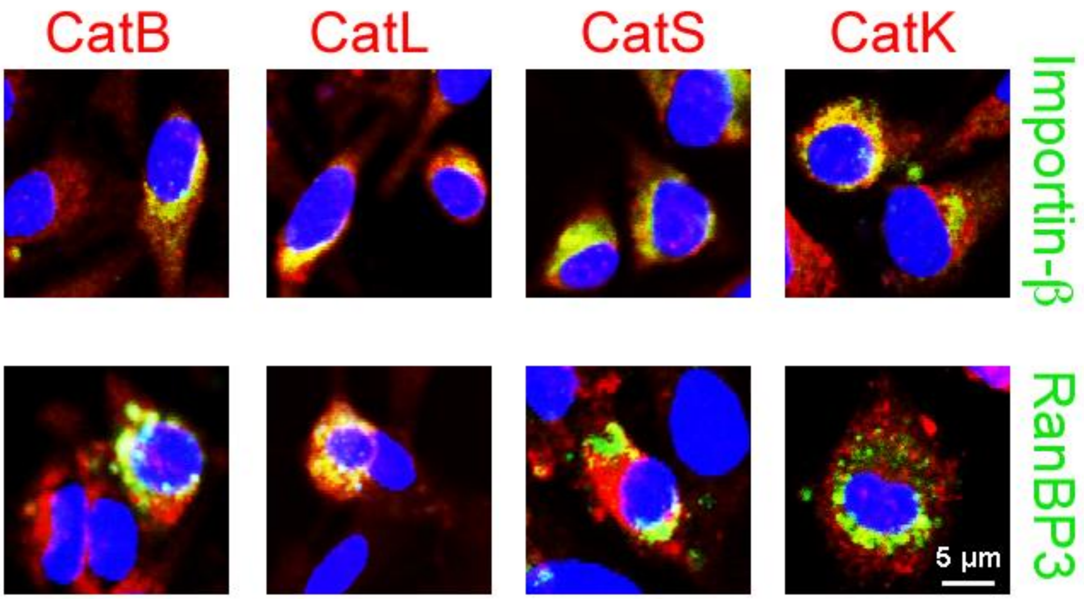

Figure S2. Cathepsin colocalization with importin-β and RanBP3 in mouse kidney TECs, related to Figure 3. Immunofluorescent double staining of different cathepsins (red) and importin-β (green) or RanBP3 (green), with nuclei counterstained with DAPI (blue) in TECs from WT mice. Scale bar: 5 μm.

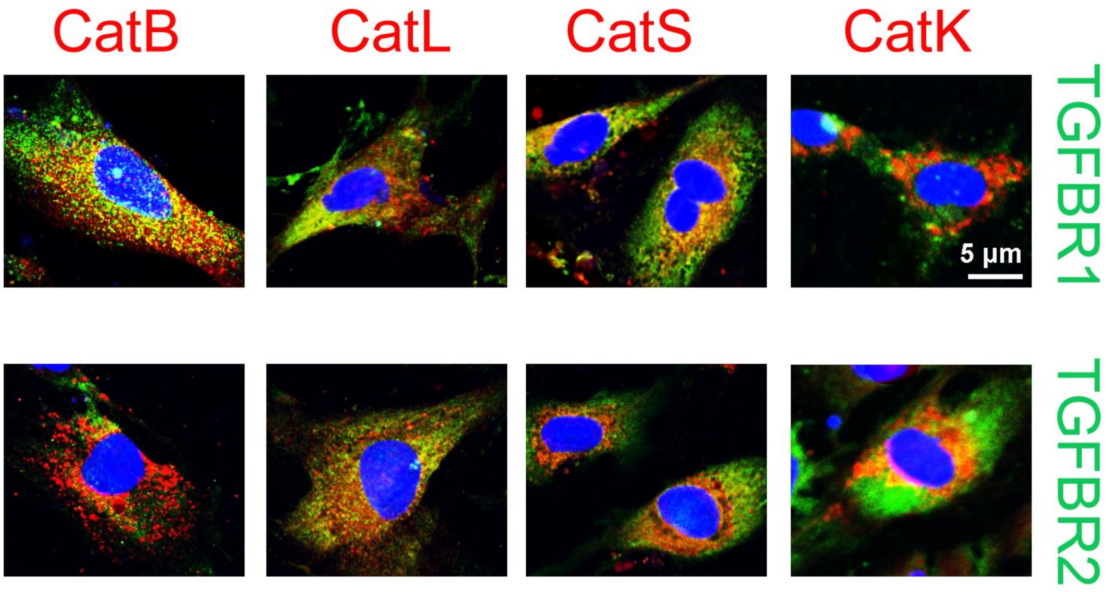

Figure S3. Cathepsin colocalization with TGF-β receptor 1 (TGFBR1) and TGF-β receptor 2 (TGFBR2) in mouse kidney TECs, related to Figure 4. Immunofluorescent double staining of different cathepsins (red) and TGFBR1 (green) or TGFBR2 (green), with nuclei counterstained with DAPI (blue) in TECs from WT mice. Scale bar: 5 μm.

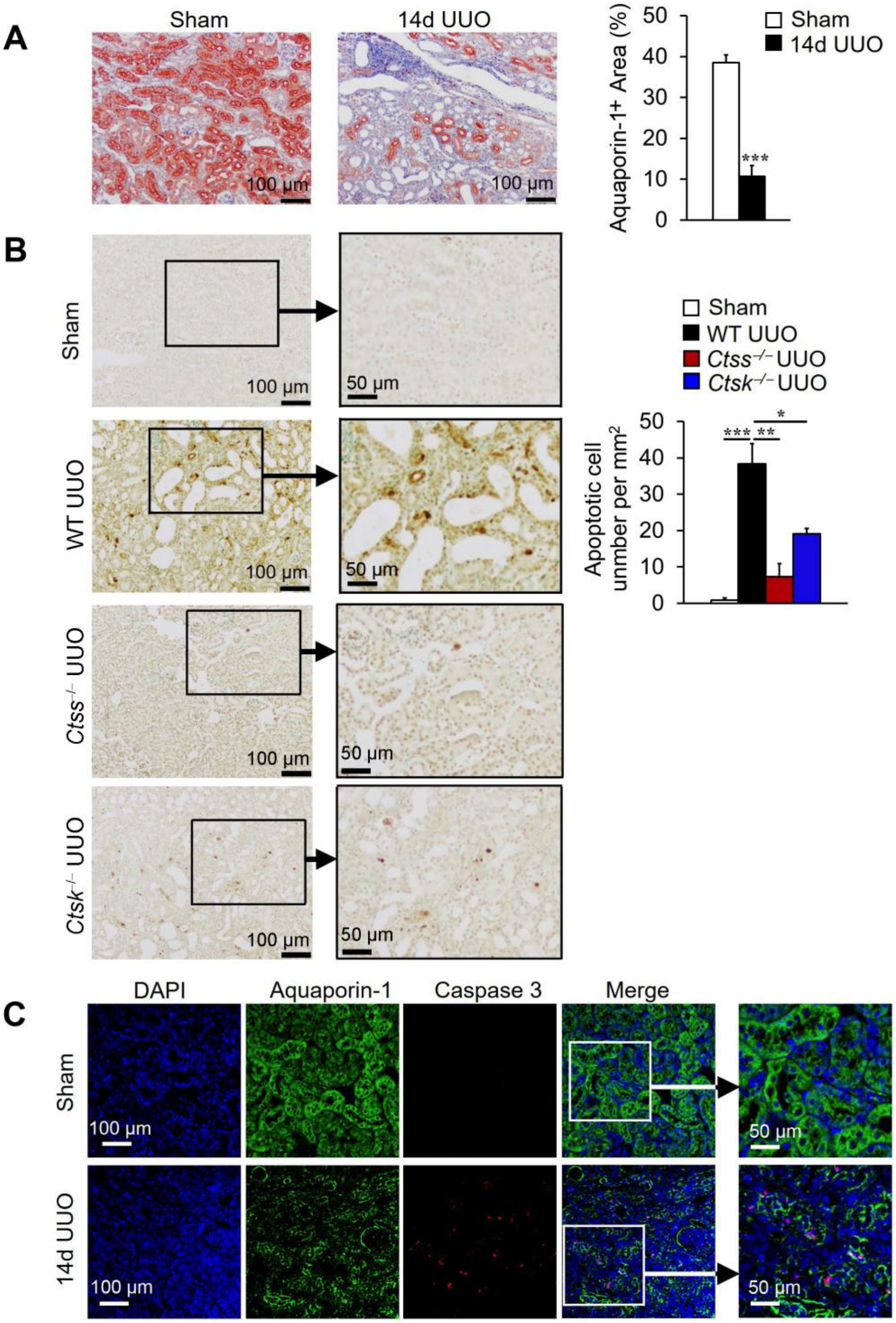

Figure S4. Kidney cortical proximal tubular damage at 14 days after UUO in wild-type mice, related to Figure 5. Immunostaining detected E-cadherin-positive area (**A**) and TUNEL-positive apoptotic cell numbers (**B**) in kidney sections from sham and UUO-injured mice as indicated. Representative images are shown to the left. (**C**) Immunofluorescent double staining of proximal tubular marker aquaprin-1 (green) and cleaved caspase 3 (red), with nuclei counterstained with DAPI (blue) in kidney section from sham and 14 days post-UUO mice as indicated. Scale: 100 µm; Inset scale: 50 µm. n=8~10 per group. *p<0.05, **p<0.01, ***p<0.001.

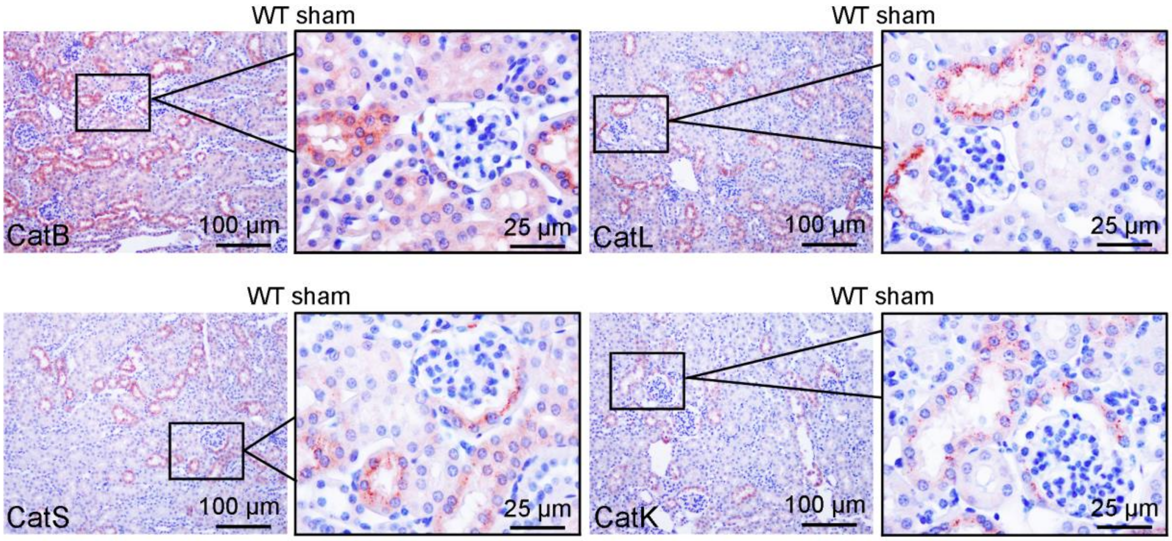

Figure S5. Immunostaining detected cathepsin expression in the tubular epithelial cells from normal mouse kidneys, related to Figure 5. No cathepsin expression was detected in the glomeruli. n=8~10 per group. Scale bar: 100 µm, inset scale: 25 µm.

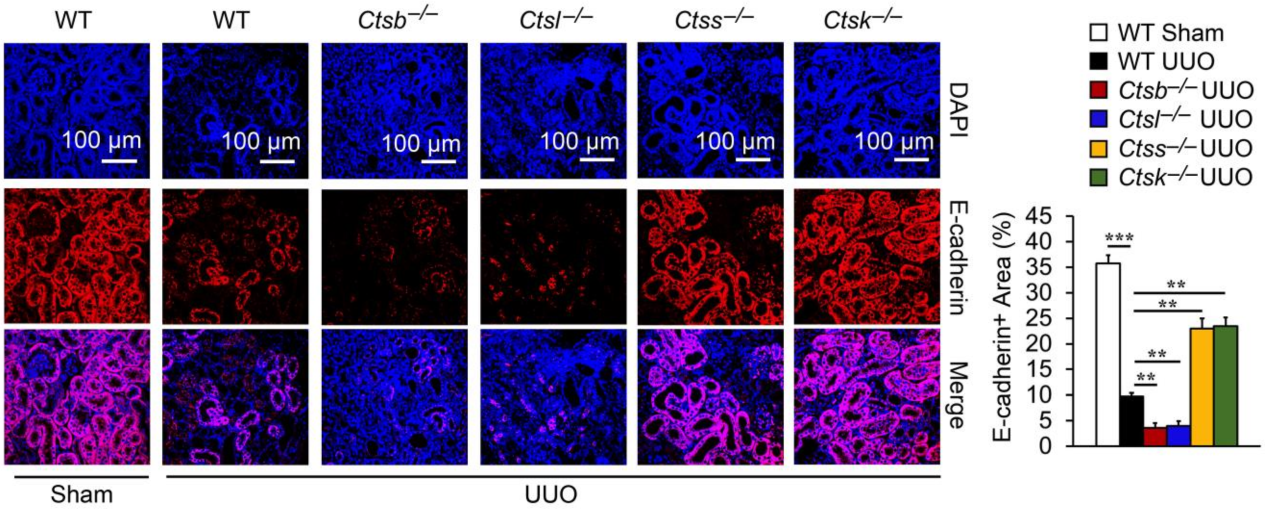

Figure S6. Immunofluorescent staining detected epithelial cell marker E-cadherin (red), with nuclei counterstained with DAPI (blue) in kidney section from different post-UUO mice as indicated, related to Figure 7. Scale bar: 100 µm. n=8~10 per group. *p<0.05, **p<0.01, ***p<0.001. Representative images are shown to the left.
